# Reconstructing foot-and-mouth disease outbreaks: a methods comparison of transmission network models

**DOI:** 10.1038/s41598-019-41103-6

**Published:** 2019-03-18

**Authors:** Simon M. Firestone, Yoko Hayama, Richard Bradhurst, Takehisa Yamamoto, Toshiyuki Tsutsui, Mark A. Stevenson

**Affiliations:** 1 Asia-Pacific Centre for Animal Health, Melbourne Veterinary School, Faculty of Veterinary and Agricultural Sciences, The University of Melbourne, Parkville, VIC 3010 Australia; 2 Viral Disease and Epidemiology Research Division, National Institute of Animal Health, National Agriculture Research Organization, Tsukuba, Ibaraki 305-0856 Japan; 3 Centre of Excellence for Biosecurity Risk Analysis, The University of Melbourne, Parkville, VIC 3010 Australia

## Abstract

A number of transmission network models are available that combine genomic and epidemiological data to reconstruct networks of who infected whom during infectious disease outbreaks. For such models to reliably inform decision-making they must be transparently validated, robust, and capable of producing accurate predictions within the short data collection and inference timeframes typical of outbreak responses. A lack of transparent multi-model comparisons reduces confidence in the accuracy of transmission network model outputs, negatively impacting on their more widespread use as decision-support tools. We undertook a formal comparison of the performance of nine published transmission network models based on a set of foot-and-mouth disease outbreaks simulated in a previously free country, with corresponding simulated phylogenies and genomic samples from animals on infected premises. Of the transmission network models tested, Lau’s systematic Bayesian integration framework was found to be the most accurate for inferring the transmission network and timing of exposures, correctly identifying the source of 73% of the infected premises (with 91% accuracy for sources with model support >0.80). The Structured COalescent Transmission Tree Inference provided the most accurate inference of molecular clock rates. This validation study points to which models might be reliably used to reconstruct similar future outbreaks and how to interpret the outputs to inform control. Further research could involve extending the best-performing models to explicitly represent within-host diversity so they can handle next-generation sequencing data, incorporating additional animal and farm-level covariates and combining predictions using Ensemble methods and other approaches.

## Introduction

Modelling the transmission network of infectious disease outbreaks is a very active research area important for informing control of transboundary and emerging infectious diseases such as Ebola haemorrhagic fever (in humans) and foot-and-mouth disease (in livestock populations). A number of dynamic models have recently been published that combine genomic and epidemiological data to reconstruct the network of who infected whom in outbreaks^1–11^. For such models to reliably inform decision-making they must be transparently validated, robust, and capable of producing accurate predictions within short data collection to inference timeframes typical of outbreak responses. Several such models have recently been assessed based on outbreak datasets simulated using very similar methods to the inferential frameworks of the models themselves^3–11^, and/or model cross-comparisons of predicted transmission networks for small clusters of infected individuals (or ‘infected premises’ in veterinary examples) such as the foot-and-mouth disease ‘Darlington cluster’ in the 2001 outbreak in the United Kingdom^7,9,10,12^. These two approaches, however, only provide a low level of ‘pseudo-validation’. The first approach is not a robust estimate of predictive performance for outbreaks arising from stochastic processes that differ from those underpinning the models themselves. The second approach does not provide a ‘gold standard’ as the true transmission network of the ‘Darlington cluster’ remains unknown, with conflicting genomic and epidemiological data. The predictive accuracy of the transmission network models thus remains unclear, particularly because it is common for different models to predict distinctly different transmission networks for the same outbreak.

Foot-and-mouth disease (FMD) is a highly contagious, acute, vesicular disease of cloven-hoofed animals (including cattle, pigs, sheep, goats and buffalo)^13^. FMD, caused by an RNA virus of the family *Picornaviridae*, is endemic in large areas of Africa, Asia and South America, and periodically causes outbreaks in previously free countries and regions. FMD is extremely resource-intensive to control due to its wide host range, contagiousness, multiple modes of transmission (direct contact, airborne and via fomites), diminished clinical signs in some species dependent on serotype (sheep and goats), and the potential for long-term carrier status in ruminants. Outbreaks in previously free countries cause severe and widespread socio-economic impacts related to the closure of international markets in livestock and animal produce on top of the direct and indirect impacts of disease control measures^14^. FMD-free countries therefore have stringent biosecurity measures in place to prevent incursions and investigate outbreaks very thoroughly.

In non FMD-endemic countries, a critical component of any outbreak response is a process known as backward and forward tracing, essentially working out who an infected individual has had contact with during their infectious period^15^. Early in the response to large-scale outbreaks, when contact-tracing is just getting underway, the implementation of appropriate interventions is time-critical^16^. At this early stage in an outbreak, when appropriate interventions can have the most impact, laboratory and field resources are often pushed to their limit^17^. Important decisions must be made under considerable uncertainty and in conditions that are continually evolving. Central questions for the outbreak investigation team are ‘who infected whom?’, ‘who will be infected next?’ and ‘how and where should we intervene?’^18^. Answering these questions in time to inform decision-makers can lead to large potential savings through better targeting who to investigate and which farms to quarantine, and/or depopulate or vaccinate.

Traditional epidemiological approaches to identifying transmission networks (contact-tracing, interviewing, laboratory sampling, typing and phylogeny of isolates), may reveal sources, points of control and provide insights into the future scale and course of an outbreak. However, obtaining detailed and accurate contact-tracing and surveillance data is not a trivial exercise early in a large outbreak. Delays in accessing data from the field and the quality of the data impact on the ability to develop meaningful predictions of disease transmission. Field investigations are resource intensive and involve a high degree of uncertainty in sources of infection and timings of unobserved events^19^. The genomics revolution has provided new tools and opportunities for tackling infectious diseases. The application of whole genome sequencing has streamlined pathogen identification in disease emergences^20^. Highly discriminatory laboratory techniques are now available for characterising the relationship between samples collected during an outbreak within appropriate timeframes and costs. Next-generation sequencing has advanced the identification of traceable differences in pathogen genomes and thereby the resolution of our understanding of disease transmission, in some cases down to the host-to-host scale^18^. This technology is now available for real-time application during outbreak responses. In this setting it is essential that epidemiological tools to guide infectious disease outbreak response are adapted to keep pace with the advances in genomics and rapid pathogen identification.

Phylogenetic trees may be topologically dissimilar to transmission trees, especially under high density sampling as would be the intention early in an incursion of an exotic animal disease or the emergence of a new infectious disease^21^. Interpreting phylogenetic proximity as epidemiological linkage can be dangerously misleading^22^. Bayesian transmission network models are the only tool presently capable of identifying the pathways of infectious disease transmission to a level of resolution required to pin-point sources. A variety of methods are available, many claiming to be a theoretical improvement on others available depending on context. A paucity of transparent multi-model comparisons unfortunately leaves it unclear how accurate they are in practice, thus reducing confidence in their interpretation and restricting their potential application in decision-support during future outbreaks. The aim of this study was to undertake a structured comparison of the performance of published transmission network models based on a set of simulated foot-and-mouth disease outbreaks so as to provide clear guidance on the application of such models to future outbreaks.

## Results

### Characteristics of the simulated outbreaks

To benchmark the performance of each transmission network modelling algorithm, a set of 100 foot-and-mouth disease outbreaks were simulated using the Australian Animal Disease Spread (AADIS) hybrid model^23^ with corresponding genomic sequences^24^ and phylogenetic trees nested within the given transmission networks^25^. The median number of infected premises (IPs) per simulation run was 92 (range: 24, 853) and the median outbreak duration was 83 days (range: 49, 323). From these model runs, six were selected for further analyses (with n = 42, 70, 98, 100, 156 and 298 IPs, respectively, referred to hereafter as runs 1 to 6). These outbreak datasets, including their genomic data and tables summarising the epidemiological variables that were available for analysis, are provided as Supplementary Materials (S1). Cattle farms were the most frequently affected with large numbers of sheep infected on smaller numbers of properties. Local spread was the predominant mode of transmission, direct animal contacts and movements through saleyards were important for disseminating infection across large areas (two to three Australian States) with infrequent airborne spread over distances >3 km.

### Comparative analyses of accuracy of inferences of transmission network models

Based on a review of the published literature, nine algorithms for integrating genomic and epidemiological data were selected for comparison^1,5–10,26,27^. We also included our own modification to the frequentist approach of Cottam *et al*.^1^ to incorporate spatial and contact-tracing data. Key attributes of these models are summarised in Table 1. Most of the modelling methods selected for comparison account for delays in sampling and tree uncertainty, make use of phylogenies, allow for unobserved hosts, and infer mutation rates and infection times for those sampled. Five of the models (‘Outbreaker’, ‘Outbreaker2’, Lau’s model, the Structured COalescent Transmission Tree Inference, ‘SCOTTI’ and ‘Phybreak’) can handle observed cases (infected premises) that have missing genomic data, a very common situation in large-scale foot-and-mouth disease outbreaks where field investigation and laboratory resources are typically severely constrained. Only four of the previously published models incorporate within-host dynamics, only two allow for multiple samples per case and only Outbreaker2 utilises contact-tracing data. The models differ in underlying likelihood estimation and the outputs returned and were only broadly comparable in terms of their accuracy of inferences of the transmission network, mutation rates and timing of first exposure. Most of the models selected for this comparison have been previously applied to infer transmission networks for foot-and-mouth disease outbreaks, with the exceptions being: ‘Sampled Ancestors’ implemented on data from a UK cluster of HIV-1 human patients, ‘BeastLier’ demonstrated through inference of a H7N7 avian influenza outbreak in the Netherlands and ‘TransPhylo’ applied on an outbreak of tuberculosis in Germany.

**Table 1:** Comparison of the features of approaches to outbreak transmission network inference from genetic and epidemiological data.

Method	Included in this study	Designed for outbreak trans-mission network inference	Package publicly available	Uses multiple samples per host	Uses exposure data	Uses sampling times	Uses phylo-genetic structure	Allows non-observed hosts	Explicitly models observed hosts missing genomic data	Uses host distance data	Uses contact-tracing data	Models within-host evolution	Allows mixed infections	Models partial trans-mission bottle-necks	Allows compart-menta-lization model	Infers mutation rates	Infers infection times
Cottam *et al*.^1^	+	+	+	−	+	+	+	−	−	−	−	−	−	−	−	−	+
Cottam *et al*. (modified)	+	+	+	−	+	+	+	−	−	+	+	−	−	−	−	−	+
Aldrin *et al*.^2^	−	−	−	−	+	−	−	−	−	+	−	−	−	−	−	−	+
Morelli *et al*.^3^	−	+	−	−	+	+	+	−	−	+	−	−	−	−	−	−^a^	+
Mollentze *et al*.^4^	−	±^b^	−	−	+	+	−	+	−	+	−	−	−	−	−	+	+
Gavryushkina *et al*.^5^ (SA)	+	+	+	−	−	+	+	+	−	−	−	−	−	−	−	+	−
Jombart *et al*.^6^ (Outbreaker)	+	+	+	−	−	+	−	+	+	±^c^	−	−	−	−	−	+	+
Lau *et al*.^7^	+	+	+	−	+	+	+	+	+	+	−	−	−	−	+	+	+
Hall *et al*.^8^ (BeastLier)	+	+	+	+	+	+	+	−	−	+	−	+	−	−	−	+	+
De Maio *et al*.^9^ (SCOTTI)	+	+	+	+	+	+	+	+	+	−	−	+	+	−	−	+	−
Klinkenberg *et al*.^10^ (Phybreak)	+	+	+	−	−	+	+	−	−	−	−	+	−	−	−	+	+
Didelot *et al*.^26^ (TransPhylo)	+	+	+	−	+	+	+	+	−	−	−	+	−	−	+	−	+
Campbell *et al*.^27^ (Outbreaker2)	+	+	+	−	−	+	−	+	+	−	+	−	−	−	−	+	+

Table based on that in^9^, updated and ordered chronologically. SA = Sampled Ancestors. ^a^The substitution rate was fixed in^3^ with mention of how to infer it. ^b^Extension of^3^ for an endemic rabies disease scenario, could be implemented on foot-and-mouth epidemics. ^c^Documentation mentions spatial model implementation is under development.

**Figure 1:**
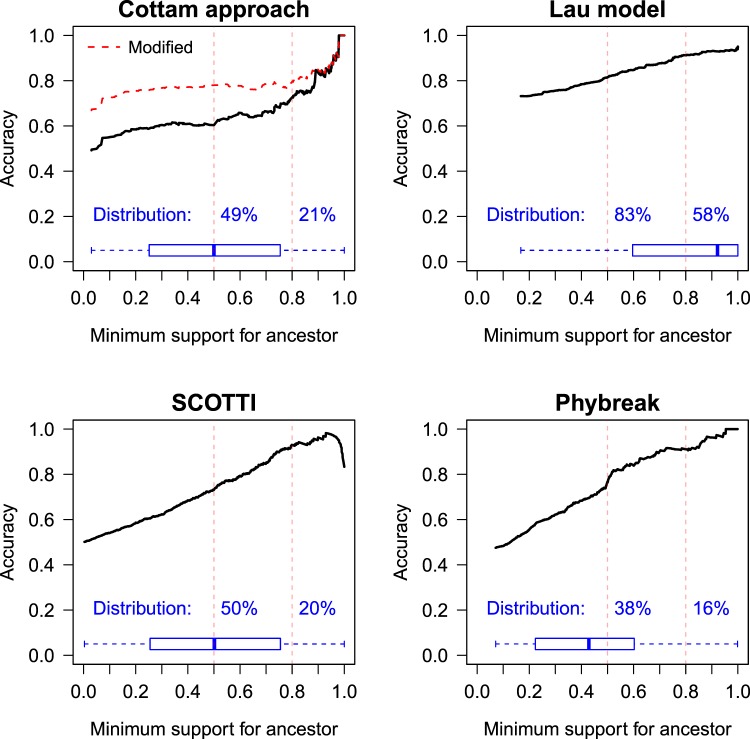
Comparison of the accuracy of inferences of transmission network models for simulated outbreaks of foot-and-mouth disease in Australia. Summarised over six simulation runs, with box-and-whiskers plots representing distribution of model support for the proposed ancestors; distribution numbers present the proportion of proposed ancestors with >50% (i.e., consensus) and >80% model support, respectively. SCOTTI = Structured COalescent Transmission Tree Inference.

**Figure 2:**
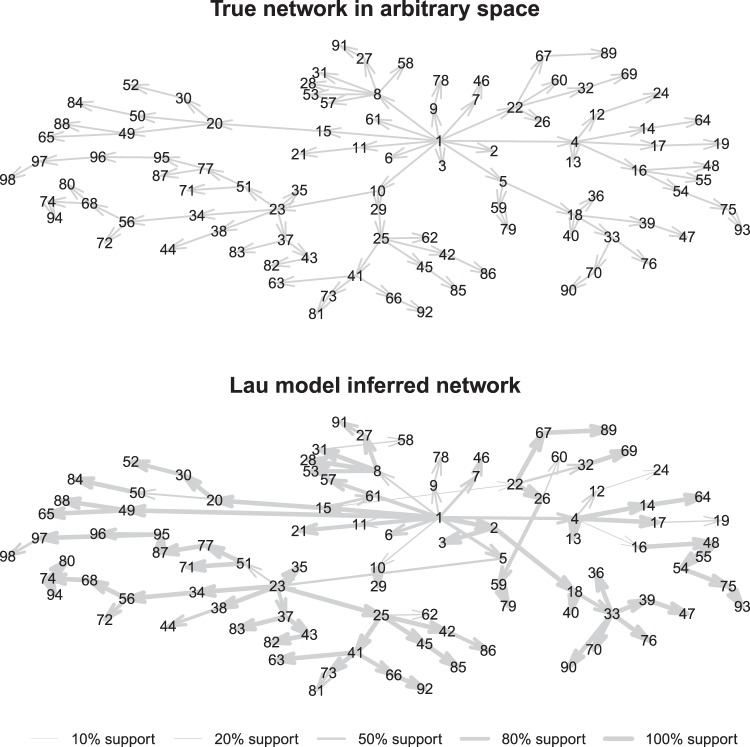
Comparison of Lau model inferred transmission network to the true network for a simulated outbreak of foot-and-mouth disease in Australia. Model support for the proposed ancestor represented by line width.

The ten modelling methods differed markedly in their ability to accurately infer the underlying transmission network (Table 2, Fig. 1). When genomic data was assumed to be available for all known infected premises Lau’s systematic Bayesian integration framework performed best with 73% of source farms correctly identified. This increased to 82% accuracy for the 83% of infected premises for which there was consensus support for a single proposed source, and increased again to 91% for the 60% of infected premises for which model confidence in their source was >0.8. The accuracy of Lau’s model was consistent across runs comprised of different numbers of infected premises (see Supplementary Materials, S3). An example of the accuracy of the inference of this model compared to the known (simulated) underlying transmission network is presented in Fig. 2. The ‘Phybreak’ model^10^ had comparable accuracy to Lau’s model for sources with high levels of model confidence, however, only 16% of infected premises had single sources with model support >0.80 from Phybreak. De Maio’s SCOTTI and the modification to Cottam’s frequentist approach were also highly accurate at inferring sources when model support was >0.80. Lau’s model identified >2.8 times as many sources with such high levels of support (Fig. 1), so overall it was the most accurate method of the ten models that were tested.

**Figure 3:**
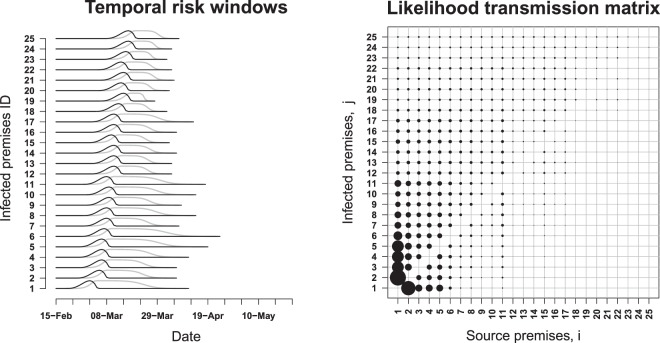
Estimated transmission windows and likelihood matrix of who infected whom based on Cottam’s frequentist approach for a simulated outbreak of foot-and-mouth disease in Australia. Black lines in temporal risk windows represent most likely period of the earliest infection of an animal on each infected premises (IP), grey lines represent estimated duration of infectiousness at the premises level, tapering as culling commences. During the most likely period when IP2 was infected, only IPs 1 to 11 were possibly infectious. Based on the likelihood transmission matrix IP2 was most likely to have been infected by IP1, and IP2 was more likely to have been infected by IP1 than vice versa. During the period when IP16 was most likely infected there were many potential sources. These likelihoods are used to rank ambiguities in the genomic parsimony network^1^.

Highly informative outputs from Cottam’s approach are presented in Fig. 3, demonstrating the estimated timing of exposure and infectious periods for infected premises, and the estimated likelihood of each possible infection source for each infected premises. Modifying Cottam’s approach to incorporate typically available spatial and contact-tracing data improved accuracy of source identification for infected premises where model confidence in the source was <80%, leading to accuracy that was overall similar to that of Phybreak and SCOTTI, and outperformed many of the highly complex Bayesian models for infected premises with lower levels of model confidence in their proposed source.

Under the more realistic scenario of genomic data only being available for 50% of observed infected premises, Lau’s model was again the most accurate of those analysed (see Table 2 and Supplementary Materials, S3). For those sources identified with high levels of model confidence, the Lau model’s inferences of the network of transmission remained very accurate (73% accuracy amongst those with >0.80 model support), however the numbers of such sources attaining model confidence decreased from 58% to 20%. Accuracy was 60% for the 47% of sources with consensus support. Phybreak was again highly accurate in inferring sources, however only for the relatively small number of infected premises for which sources were identified with consensus support (only 50 of 764 inferred sources had consensus support).

**Table 2:** Comparison of the accuracy of inferred transmission networks, summarised over six simulated outbreaks of foot-and-mouth disease in Australia, by transmission network model.

Algorithm	Accuracy^a^		
	Overall (%)	>50% support (%)	>80% support (%)
**Genomic data available for all known infected premises**			
Cottam *et al*.^1^	373/758^b^ (49)	231/369 (63)	115/158 (73)
Cottam *et al*. (modified)	507/758^b^ (67)	288/369 (78)	126/158 (80)
Gavryushkina *et al*.^5^ (SA)	123/511^c^ (24)	72/249 (29)	47/102 (46)
Jombart *et al*.^6^ (Outbreaker)	294/764 (38)	282/665 (42)	241/490 (49)
Lau *et al*.^7^	559/764 (73)	519/636 (82)	406/445 (91)
Hall *et al*.^8^ (BeastLier)	118/764 (15)	66/382 (17)	31/157 (20)
De Maio *et al*.^9^ (SCOTTI)	383/764 (50)	282/382 (74)	144/155 (93)
Klinkenberg *et al*.^10^ (Phybreak)	363/764 (48)	222/290 (77)	112/123 (91)
Didelot *et al*.^26^ (TransPhylo)	29/764 (4)	25/516 (5)	8/177 (5)
Campbell *et al*.^27^ (Outbreaker2)	269/764 (35)	214/470 (46)	140/276 (51)
**Genomic data missing for 50% of known infected premises**			
Jombart *et al*.^6^ (Outbreaker)	55/764 (7)	41/214 (19)	32/119 (27)
Lau *et al*.^7^	319/764 (42)	212/356 (60)	112/154 (73)
De Maio *et al*.^9^ (SCOTTI)	93/382^d^ (24)	73/193 (38)	37/78 (47)
Klinkenberg *et al*.^10^ (Phybreak)	132/764 (17)	36/50 (72)	5/7 (71)
Campbell *et al*.^27^ (Outbreaker2)	21/764 (3)	4/50 (8)	0/20 (0)

IP = infected premises; SA = Sampled Ancestors. Detailed results per model run provided in Supplementary Materials (S3).

^a^Accuracy was defined as the proportion of IPs for which the model-predicted most likely source (highest likelihood or most posterior support) was the true source. The denominator for accuracy at > 50% (i.e., consensus) and >80% support includes only those IPs for which the model-predicted most likely source attained that level of likelihood or posterior support. ^b^In each run, the root was fixed based on best guess. ^c^Not all IPs detected as having sampled ancestors. ^d^SCOTTI only outputs proposed ancestors for those IPs with genomic data available.

**Figure 4:**
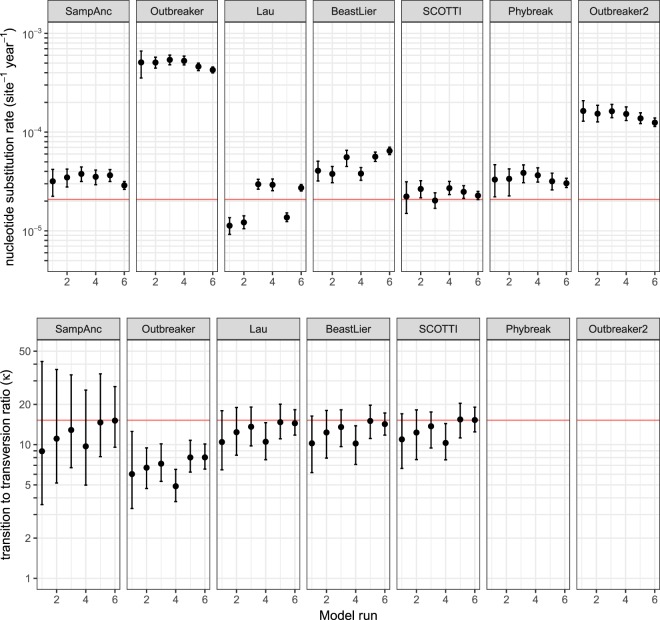
Comparison of the accuracy of inferences of mutation rates for six simulated outbreaks of foot-and-mouth disease (FMD) in Australia, by transmission network model. The red reference lines represent the true (simulated) value of each parameter. The Phybreak model assumes a Jukes-Cantor nucleotide substitution model under which the ratio of transitions to transversions (TrTv) is not inferred. Outbreaker2 also does not output TrTv. SampAnc = Sampled Ancestors model; SCOTTI = Structured COalescent Transmission Tree Inference.

Of the seven studied models that infer mutation rates for the transmitted virus, SCOTTI consistently provided the most accurate inference of the simulated molecular clock rate (*μ*) of 2.078 mutations × 10^−5^ site^−1^ day^−1^ (mean bias + 15%; 95% highest posterior density [HPD] −5%, +39%), see Fig. 4. Lau’s model consistently provided inferences of the clock rate close to and around the true value (mean bias −1%; 95% HPD: −12%, +12%). The Sampled Ancestors, Phybreak and BeastLier models all marginally overestimated the mutation rates (mean biases of +64%, +64% and +235%, respectively), whereas the Outbreaker and Outbreaker2 models markedly overestimated the mutation rate by means of 24-fold (95% HPD: 20.7, 17.1-fold) and 7-fold (95% HPD: 6.1, 8.5), respectively, across the 6 model runs. SCOTTI, Lau’s model and BeastLier all provided accurate and precise inference of the transition to transversion ratio (*κ*) (Fig. 4). Sampled Ancestors’s inference of *κ* was close to the true value of 15.22, however there was considerable uncertainty in the inference. Outbreaker underestimated κ by 55% (95% HPD: 35, 67%), largely due to underestimation of the rate of transitions (*μ*_1_). The Phybreak model assumes the Jukes-Cantor nucelotide substitution model under which only a single mutation rate is inferred, so the ratio of the rate of transitions (*μ*_1_) to the rate of transversions (*μ*_2_) is not a model output. The present implementation of Outbreaker2 only infers a single mutation rate (*μ*).

**Figure 5:**
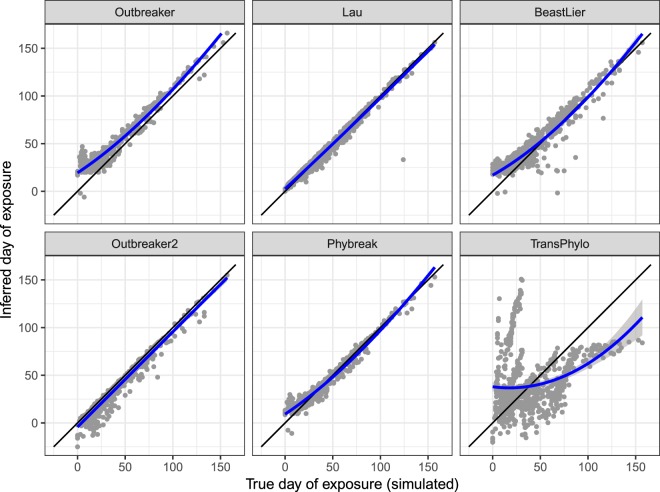
Comparison of the accuracy of inferences of timing of exposure for infected premises in six simulated outbreaks of foot-and-mouth disease (FMD) in Australia, by transmission network model. Departures above the black reference line are premises with inferred day of exposure later than their true (simulated) day of exposure. LOESS smoothed line in blue with standard error of prediction.

Of the Bayesian models that outputted inferred timing of exposure for each individual premises, Lau’s model provided the least biased inference of timing of exposure (Fig. 5) with overall coverage of 70% and median bias of +1 days (95% HPD: −8, +5 days); see Supplementary Materials S3 for detail on coverage and bias by model run. Klinkenberg’s Phybreak model had a higher overall coverage at 80% and highly comparable mean bias of +1 days (95% HPD −10, +16 days), however it had less certainty in the inference with inferred timings of infection later than actual dates at the start and end of the simulated epidemics (see Fig. 5). Outbreaker2 provided a relatively unbiased inference of timing of exposure over the whole set of infected premises with high overall coverage of 87%, however this was confounded by the large uncertainty in the inferred timings of infection (median bias −2 days; 95% HPD: −22, +1 days) and the inferred timings appeared mostly earlier than the simulated values on visual inspection. Beastlier had an overall coverage of only 24 and tended to provide marginally later inferences than the true values early in the simulated outbreaks, rapidly improving as the outbreaks progressed (LOWESS estimator in Fig. 5 departing above the reference line). Inference of timing of exposure by Jombart’s Outbreaker was consistently biased (overall median 11 days later than the true value; 95% HPD: −2, +29 days), irrespective of model run or time elapsed in the outbreak. Didelot *et al*.’s TransPhylo model was largely inaccurate in the inference of timing of infection with coverage of only 17%.

### Sensitivity analyses

In a ‘non-spatial’ comparison run (with the spatial coordinates of each infected premises randomised) accuracy of prediction of sources decreased for the modified Cottam approach and BeastLier, whereas the Lau model’s predictive accuracy was robust to the lack of a spatial signal in transmission. As expected, predictive accuracy for the seven models that do not include a spatial aspect in their inference were highly comparable to baseline in the ‘non-spatial’ comparison run (see Supplementary Materials, Table S3.3). In a ‘fast clock’ comparison run (with a 10-fold increased rate of nucleotide substitutions), the modified Cottam approach demonstrated improved predictive accuracy for sources with lower than consensus model support. The predictive accuracy of SCOTTI was markedly reduced in the ‘fast clock’ scenario. Predictive accuracy of sources proposed by the Lau model were robust to the fast clock scenario, as were those of BeastLier, Phybreak, TransPhylo. Model support was higher for sources proposed by Outbreaker and Outbreaker2 under the ‘fast-clock’ scenario whilst predictive accuracy remained highly comparable to baseline.

## Discussion

This analysis presents the first structured comparison of available transmission network modelling methods that attempt to infer ‘who infected whom’ in outbreaks. The strengths and limitations of nine publicly available models were identified based on inferences of a set of six independent simulations of a hypothetical foot-and-mouth disease outbreak in Australia. Each model has various strengths and weaknesses, and each provides different outputs tailored to the purpose for which it was developed. Many of the differences observed in model performance may be explained by the amount of epidemiological data used and the entirely different approach each takes to infer the transmission network, including in each case highly sophisticated specifications of likelihood or pseudo-likelihood functions based on assumptions of the systems under inference.

The model developed by Lau and colleagues^7^ consistently outperformed all others in terms of accuracy of identifying the sources of infection for each infected premises, model support for each inferred source, and timing of first exposure of each infected premises. SCOTTI performed the best for inference of genomic substitution rates. In inferring the transmission tree, Lau’s model incorporates inferences of the genomes present on infected premises in the transmission network at multiple points in time, a flexible spatial transmission kernel representing the proximity of infected premises, and epidemiological parameters including the latent and infectious periods (as sojourn times in the compartments of the susceptible-exposed-infected-recovered process). Genomes can be inferred with Lau’s model for known hosts that are unsampled, a common scenario in the highly resource-constrained environment of large infectious disease outbreak in a highly susceptible population. Lau’s model was understandably less accurate when run on outbreak simulations where genomic data was only available for 50% of observed infected premises. Nevertheless, Lau’s model was 60% accurate for the 356 of 764 (46%) inferred sources that attained consensus support. In comparison, Phybreak had higher accuracy (72%) when considering only those sources with consensus support. A limitation of Phybreak’s inference is that only 50 of 764 (6.5%) inferred sources considered attained consensus support. The three other models that also make transmission network inferences where there are unsampled cases (SCOTTI, Outbreaker and Outbreaker2) were all a lot less accurate.

Cottam’s original frequentist approach outperformed many of the more sophisticated Bayesian models, especially once modified to incorporate spatial relationships between infected premises and available contact-tracing data. The original formulation was able to accurately estimate a lot of the major structure of the transmission network and provides useful visualisation of the temporal epidemiological aspect of the outbreak under investigation. Lower accuracy of inference for sources of infected premises with lower likelihood was partly overcome by incorporating spatial relationships and available contract-tracing data. In outbreaks of foot-and-mouth disease and other exotic, infectious animal diseases in previously free countries, considerable resources are invested in accurately collecting such data within timeframes that can inform further field investigations and response activities. Such data are often imperfect, as represented in the present analysis by ‘true’ and ‘false traces’ that were considered in terms of likelihood based on other available epidemiological data, particularly estimated timing of onset of clinical signs and infectious period on the farms sending and receiving potentially infected animals. The method required extensive coding in R to automate the process of transcribing and capturing the considerable inherent ambiguities in the genomic parsimony tree exported from the TCS program^28^. Cottam’s method proved more difficult to implement than those presently available in BEAST^29,30^ (i.e., SCOTTI, Sampled Ancestors, BeastLIER) and the R statistical package^31^ (i.e., Phybreak, Outbreaker, Outbreaker2 and TransPhyloR), and only seems feasible for relatively small outbreak datasets. The Lau model is presently coded in C++ and although relatively straightforward to compile and run it could be made more accessible by recoding it for use as an R package.

Each method provided imperfect inference of the transmission network. Therefore, when implemented on the same benchmark outbreak, each model produced markedly different inferences of the transmission network. On the surface, such differences hinder interpretation and application in decision-making. However, when model support for each proposed ancestor was considered each model demonstrated improving accuracy as support increased, with considerably different patterns in this correlation. This makes sense considering that the models gain support for their proposed sources based on different likelihood estimation algorithms. In practice, where the models agree on a proposed source and that it has a high level of model support, then confidence can be increased that this is the source of infection for a given node in the transmission tree. Differences in model outputs may also be useful. An area for further research is in the application of Ensemble methods, such as weighted Bayesian model-averaging, given these have often been shown to perform better than any single model^32^.

The findings of the present methods comparison are consistent with publications that first describe the models assessed. In a direct comparison SCOTTI demonstrated higher accuracy than Outbreaker in inferring the transmission tree on simulated outbreak data^9^. Phybreak had similar accuracy as reported here at high levels of model support, including simulations with molecular clocks with rates comparable to those of the simulations in the present analysis^10^. Some methods do not use the available onset of clinical signs (‘exposure data’), i.e., Sampled Ancestors, Outbreaker and Phybreak, rather being based on generation time and sampling interval estimation. This can lead to discrepancy in inference of the timing of infection.

It is not possible for transmission network models to capture all of the inherent complexity of an outbreak such as a foot-and-mouth disease incursion into a previously naïve animal population. Many unobserved biological processes operate at the farm level, the individual animal level and within the individual host, including complex evolutionary processes such as transmission bottlenecks, recombination of genomic material, and reassortment (in the case of influenza viruses). None of the models presented here can explicitly represent all the organisational hierarchies and complexities present in real biological systems (i.e., multiple viral pseudo-species evolving in multiple animals per farm across a landscape). Understandably, capturing all such detail is unlikely to be necessary, especially in the two main settings where such tools are intended to be used to inform decision-support: in real-time early in unfolding outbreaks and retrospectively to provide comprehensive assessments of what transpired to inform preparedness for future events.

The validity of this comparison study depends on the plausibility of the simulated outbreaks, genome sequences and phylogenies. The Australian Animal Disease Spread (AADIS) hybrid model^23^ is the most sophisticated model available with the specific purpose of simulating outbreak scenarios like those developed here^33,34^. This model, and its predecessor AusSpread^35^, have been extensively tested in model comparison studies^36–38^ and is also presently being applied in disease preparedness scenario development for the Australian Department of Agriculture and Water Resources^17,34,39,40^. The comparison exercise reported in this paper was so resource intensive that only six simulation runs could be inferred by each of the 10 modelling algorithms tested, a similar number to the only other comparable study^25^, although that comparison study had different objectives. The coalescent model represented the underlying within-host genomic diversity as a constant effective population size. This could favour the performance of models that happen to have a similar underlying nucleotide substitution models such as the Lau model, Outbreaker and Outbreaker2 over those that implement more complex genomic inferences (such as SCOTTI, Phybreak, TransPhylo, BeastLier and Sampled Ancestors). Resource constraints limited the range of scenarios that could be tested. In a sensitivity analysis designed to test the influence of two aspects of the underlying simulations, predictive accuracy of the Lau model appeared robust to substantial changes in spatial signal underlying transmission and on clock rate. Predictive accuracy of the modified Cottam method appeared to depend on spatial parameterisation and clock rate, SCOTTI’s accuracy depended on clock rate, as did the level of model support outputted by Outbreaker and Outbreaker2. It is therefore not advised to generalise the findings of the present analysis with respect to these models too widely without further testing. Past comparisons have largely relied on data simulated in similar frameworks to the models under investigation or been conducted on real data from outbreaks where it is impossible to truly know the transmission network and therefore not possible to objectively compare model performance. This analysis has advanced as far as practical the level of formal comparison of transmission network models by simulating outbreak data in a framework completely different to the Bayesian inferential frameworks evaluated.

## Conclusions

The findings of this comparison study point to which models might reliably reconstruct future outbreaks and how to interpret the outputs to inform control. Each model assessed had its own strengths related to the purpose for which it was developed, and limitations related to its assumptions. These should be kept in mind when choosing a method, or methods, to implement based on a given context. The model developed by Lau and colleagues was consistently the most accurate in inference of the transmission tree. The Structured Coalescent Transmission Tree Inference (SCOTTI) provided a highly accurate inference of the molecular clock rates. Further research could involve extending the best-performing of these models to incorporate additional animal and farm-level covariates, represent within-host diversity so they can make most use of next-generation sequencing data, and to develop combined predictions using Ensemble methods and other approaches.

## Materials and Methods

### Simulation of epidemic spread and genomic data

The Australian Animal Disease Spread (AADIS) hybrid model was used to simulate 100 foot-and-mouth disease outbreaks using the baseline configuration, described in detail by Bradhurst *et al*.^23^, with movement restrictions and a stamping out only policy (i.e., no vaccination) from the point of outbreak detection fixed at 21 days after seeding on a large pig farm in central Victoria (infected premises 1, IP1). From these model runs, five were randomly selected representing the range of total infected premises (IPs) for all of the simulated outbreaks. One further run was selected with close to the total number of infected premises in the 2010 outbreak of foot-and-mouth disease in Miyazaki Prefecture of Japan (with *n* = 298 IPs). These six simulated outbreaks were mapped and plotted in R version 3.4.1^31^ using the contributed packages epiR^41^, statnet^42^ and sp^43^.

The origin of the outbreak was designated with the 7667 nucleotide whole genome consensus sequence (O/JPN/2010-6/1 S) sampled from the first farm presumed to be infected in the 2010 outbreak of foot-and-mouth disease in Miyazaki Prefecture of Japan^44^. Based on AADIS model outputs (date of exposure, date of diagnosis and the edge-list of the known transmission network), we simulated a sequence to seed the primary infected premises (IP1) assumed to have been infected 30 days after sampling of the sequence O/JPN/2010-6/1 S, then forward simulated sequences for each of the subsequent infected premises (IP2, …, IP*n*) based on the known transmission trees for these simulated outbreaks and these premises’ simulated days of exposure and sampling.

In densely sampled outbreaks, such as the context of this study (foot-and-mouth disease detected in a previously free country), coalescent times and transmission times can differ markedly, leading to important differences between the transmission network and the phylogenetic tree nested within it^45^. Following^25^, we simulated phylogenetic trees nested within the given transmission networks with VirusTreeSimulator (https://github.com/PangeaHIV/VirusTreeSimulator; last accessed 3 December, 2018) then SeqGen version 1.3.3^24^ for Monte Carlo simulation of molecular sequence evolution along the simulated phylogenetic trees. Based on empirical observations of model fit and parameters from the 2001 outbreak of FMD in the UK^1,12^ and the 2010 outbreak of FMD in Japan^44^, we assumed the HKY model^46^ with a rate of nucleotide substitutions of 2.168 × 10^−5^ changes per site per day, with a ratio of 7.61 transitions to transversions, empirically estimated nucleotide frequencies (of *π*_*A*_ = 0.253, *π*_C_ = 0.282, *π*_*G*_ = 0.257, *π*_*T*_ = 0.208), mutations assumed to occur independently along the sequence owing to little selective pressure, and no recombination, insertions or deletions within the short time-frames of the epidemics under consideration. Within-farm diversity was modelled with a within- and between-host neutral coalescent model assuming a constant effective population size of 100 and that the bottleneck at transmission was complete. This model is described in detail elsewhere^11^. Only a single sample was considered per IP, given that sequencing of virus from different animals from the same farm in the UK 2001 outbreak indicated very limited within-farm sequence variability^12^.

Simulated outbreak datasets including genomic data are provided as Supplementary Material (S1) along with epidemic curves, maps and tables of simulated variables available for each infected premises. Only those data considered likely to be available in near real-time in future outbreaks were used to infer transmission networks.

### Transmission network modelling algorithms

Based on a review of the published literature, nine models were selected for comparison in the present study^1,5–10,26,27^ on the basis of their having being developed specifically for inferring the transmission network of outbreaks based on epidemiological and genomic data, and either their source code or executable packages being openly available for implementation. We also modified the only frequentist algorithm^1^ to include spatial and contact-tracing data. Detailed methods of their implementation in the present study are presented in Supplementary Materials (S2). Three other published models were identified and considered for inclusion in this study but were not used due to either executable packages or source code not being publicly available and/or they were not developed specifically for outbreak transmission network inference^2–4^.

### Comparative analyses of accuracy of inferences

To compare the ‘accuracy’ of each benchmarked method, we estimated the proportion of IPs whose true source was correctly identified across all six model runs. For methods that provided estimates of the support (confidence or posterior probability) in the proposed source, this was based on the potential source with the highest level of support, and we separately calculated the accuracy of the most likely tree for each method considering only those sources with consensus and >0.80 support. For methods that estimated other parameters, such as mutation rates and timing of infection, estimates and coverage were compared to the true values.

In the context under consideration, an FMD outbreak occurring in a previously-free country, all IPs would be expected to be observed by the end of the outbreak, or in real-time application, methods could be implemented to infer undetected infections^19^. However, not all such premises would be expected to be sampled. For all methods that could handle missing sequence data from observed cases, the analysis was repeated assuming that a more realistic subset of only 50% of the IPs had whole genome sequences available. This sampling density reflects expected practice in future outbreaks considering that 104 sequences were available from 292 infected premises in the 2010 FMD disease outbreak in Miyazaki, Japan^44^.

### Sensitivity analyses

To test for the influence of key underlying simulation modelling assumptions on the accuracy comparison, and general applicability of this analysis, one of the moderately sized simulated outbreaks (run 3 with n = 98 IPs) was reparametrized in two ways. Firstly, a ‘non-spatial’ run was generated by randomising the spatial coordinates of each infected premises, thus eliminating the spatial signal in transmission. Then, separately, a ‘fast clock’ run was generated by increasing the rates of transitions and transversions by a factor of 10, leaving the generation time unchanged. All inferences were repeated on these two modified datasets and model outputs again compared.

## Supplementary information

Supplementary Information

## Data Availability

All data generated or analysed during this study are included in this published article (and its Supplementary Information files).
