# Supplementary material for: Reconstructing foot-and-mouth disease outbreaks: a methods comparison of transmission network models
Source: Sci Rep. 2019 Mar 18;9:4809. doi: 10.1038/s41598-019-41103-6 (PMC6423326; doi:10.1038/s41598-019-41103-6)
Supplement: Supplementary file 1 — Supplementary Information [file 41598_2019_41103_MOESM1_ESM.pdf]

Supplementary Materials:

Reconstructing foot-and-mouth disease outbreaks: a methods comparison of transmission network models

Simon M. Firestone[1]*, Yoko Hayama[2], Richard Bradhurst[3], Takehisa Yamamoto[2], Toshiyuki Tsutsui[2], Mark A. Stevenson[1]

[1] Asia-Pacific Centre for Animal Health, Melbourne Veterinary School, Faculty of Veterinary and Agricultural Sciences, The University of Melbourne, Parkville, VIC 3010, Australia

[2] Viral Disease and Epidemiology Research Division, National Institute of Animal Health, National Agriculture Research Organization, Tsukuba, Ibaraki 305-0856, Japan

[3] Centre of Excellence for Biosecurity Risk Analysis, The University of Melbourne, Parkville, VIC 3010, Australia

* Corresponding author: simon.firestone@unimelb.edu.au

S1: Outbreak datasets and descriptive statistics

Six simulated AADIS [1] outbreak datasets with genomic data simulated using VirusTreeSimulator [2] and SeqGen [3], and associated maps, epidemic curves, known transmission networks and phylogenetic trees.

Available as online repository: https://doi.org/10.26188/5c0a6973e4d43

List of files

AU_infdate.run1.gif	Animated GIF of simulated outbreak.
AU-FMD-sequsim.run1-6.fasta	FASTA files of simulated foot-and-mouth-disease virus (FMDV) sequences, by model run.
epi.tab.run1-6.csv	Epidemiological data for simulated infected premises. See Table A below for metadata.
FMD-AU-arbitrary.space.run1-6.pdf	Simulated transmission networks in arbitrary space.
FMD-AU-epicurve.run1-6.pdf	Simulated epidemic curves.
FMD-AU-map.run1-6.pdf	Simulated epidemic maps.
FMD-AU-phylo.run1-6.pdf	Simulated phylogenetic trees.
FMD-AU-time-tree.run1-6.pdf	Simulated transmission networks in arbitrary space, with time dimension.

Table S1.1: Variables available for each infected premises (IP) in six simulated outbreaks of foot-and-mouth disease (FMD) in Australia.

Variable	Description
Herd ID*	Unique identifier
Geocoordinates*	Latitude and longitude in GDA94 coordinate reference system
Herd type*	Predominant type: beef intensive, beef extensive, dairy cattle, mixed beef, feedlot, sheep, mixed sheep, pigs small, pigs large or smallholder (mixed)
Number of animals*	For FMD susceptible species only
Day of infection	Of the first animal infected on this premises
Day of onset*	Onset of clinical signs in the earliest animal detected on this premises.
Day of diagnosis*	Also assumed to be earliest day of sampling for those farms assumed to have sequence data available
Reason diagnosed*	Index premises, suspect premises, traced premises, at-risk premises, dangerous contact premises[a]
Day culling started*	Or estimated last day infectious if not culled whilst infectious.
Source herd ID	As this is simulated data the true source is known.
Infection pathway	Seeding, direct (i.e. direct contact or animal movement), indirect (i.e. transmission on fomites), through saleyard movement, local spread[b] or airborne.
Contact-tracing history*	Where available from and/or to this herd (i.e. backwards- and forwards-tracing data) collected from simulated surveillance activities which can be ‘true traces’ (that identify the actual transmission to or from this IP) and ‘false traces’ (that identify other links that did not lead to transmission events).

* Variables used for inference, i.e. those that would be likely to be available in near real-time during a future outbreak.

[a] Defined following the Australian Veterinary Emergency Plan [4].

[b] Defined as spread between IPs within 3 km of each other by more than one possible mode of infection: the majority will be from either aerosol spread over short distances between animals (particularly where premises boundaries are contiguous) or contamination in the area near an IP, resulting in infected material on roads or other common facilities. Airborne spread by plumes of virus over greater distances was modelled separately [5].

Table S1.2: Details of six simulated outbreaks of foot-and-mouth disease (FMD) in Australia used to benchmark transmission network modelling methods.

Simulation run	Outbreak duration (days)	Total no. of IPs	States affected (IPs)	Farm types (IPs)	Animals affected (by farm type)	Modes of infection[a] (IPs)
1	81	42	NSW (2)	Dairy cattle (13)	4,236	Seed (1)
			VIC (40)	Beef cattle (19)	1,631	Direct (3)
				Feedlot cattle (1)	3,880	Saleyard (8)
				Sheep (8)	10,169	Local spread (30)
				Pigs (1)	3,209	
2	92	70	NSW (24)	Dairy cattle (16)	4,953	Seed (1)
			VIC (46)	Beef cattle (33)	4,520	Saleyard (8)
				Sheep (20)	19,744	Indirect (6)
				Pigs (1)	3,209	Local spread (55)
3	88	98	NSW (6)	Dairy cattle (22)	7,988	Seed (1)
			VIC (92)	Beef cattle (37)	5,209	Direct (11)
				Feedlot cattle (1)	204	Saleyard (17)
				Sheep (29)	37,248	Indirect (3)
				Pigs (6)	5,599	Local spread (63)
				Smallholder (3)	19	Airborne (3)

Continued over page.

IP = infected premises; NSW = New South Wales; QLD = Queensland; VIC = Victoria; Beef cattle estimates include mixed beef farm types, sheep estimates include mixed sheep farm types; local spread includes aerosol transmission within 3 km, airborne transmission defined as over distances >3 km.

Table S1.2 (cont.): Details of six simulated outbreaks of foot-and-mouth disease (FMD) in Australia used to benchmark transmission network modelling methods.

Simulation run	Outbreak duration (days)	Total no. of IPs	States affected (IPs)	Farm types (IPs)	Animals affected (by farm type)	Modes of infection[a] (IPs)
4	87	100	NSW (2)	Dairy cattle (44)	12,554	Seed (1)
			QLD (2)	Beef cattle (40)	4,499	Direct (4)
			VIC (96)	Feedlot cattle (2)	40,436	Saleyard (6)
				Sheep (12)	14,107	Indirect (1)
				Pigs (1)	3,209	Local spread (85)
				Smallholder (1)	5	Airborne (3)
5	82	156	NSW (22)	Dairy cattle (32)	9,348	Seed (1)
			VIC (134)	Beef cattle (75)	9,471	Direct (9)
				Feedlot cattle (7)	62,276	Saleyard (18)
				Sheep (36)	44,197	Indirect (13)
				Pigs (1)	3,209	Local spread (112)
				Smallholder (5)	23	Airborne (3)
6	170	298	NSW (17)	Dairy cattle (83)	22,206	Seed (1)
			VIC (281)	Beef cattle (156)	19,982	Direct (7)
				Feedlot cattle (4)	7,938	Saleyard (29)
				Sheep (40)	40,746	Indirect (9)
				Pigs (12)	5,375	Local spread (252)
				Smallholder (3)	23	Airborne (0)

IP = infected premises; NSW = New South Wales; QLD = Queensland; VIC = Victoria; Beef cattle estimates include mixed beef farm types, sheep estimates include mixed sheep farm types; local spread includes aerosol transmission within 3 km, airborne transmission defined as over distances >3 km.

S2: Detailed methods on implementation of transmission network algorithms

Cottam's frequentist approach [6]

Maximum parsimony networks of the genetic relationship between sequences from each infected premises (IP) were constructed using TCS version 1.21 [7] and exported into the R statistical package version 3.4.1 [8] using the igraph library [9] then manipulated with a purpose-built script to enable further analyses. When inferring the transmission tree, these parsimony networks have a high degree of inherent ambiguity. The most likely tree was selected by ranking the trees based on the temporal aspect of available epidemiological data. Essentially, for each IP, at each point in time, the likelihood that animals on the farm were first infected and also the likelihood that animals are in their infectious period is estimated (based on observed data: Day of onset, First and Last days of culling) implementing formulae as presented in [6]. This enables estimation of the likelihood that each potential source was indeed the source of infection for each IP, except for any root nodes which must be specified if unclear from the parsimony network. As the temporal likelihoods of links are typically very low and severely positively skewed, to make for meaningful interpretation of accuracy over the range of likelihoods, the likelihoods were non-parametrically rescaled between 0 and 1. Following [10], whenever TCS generated complex multilinked trees, the shortest routes through these trees (which did not involve back-mutations) was selected. This approach does not have an explicit means to account for non-observed hosts or observed hosts lacking genomic data, however these may be considered as potential sources for links with very low estimated likelihood for all known sources. The full process took approximately 1 hour to run, the TCS component and likelihood ranking being quick and straightforward, however scripting the process to define all of the possible transmission networks for ranking based on the TCS parsimony network was laborious and error-prone.

Modification of Cottam's frequentist approach

Extending the approach of [6], originally developed specifically for FMD outbreaks in previously free countries, to incorporate spatial relationships and contact-tracing data in a data structure mimicking that expected to be available in such outbreaks affecting farm animal populations [11,12], involved firstly selecting only those 'possibly infectious traces' from or to each IP where an animal movement occurred:

- between premises detected as infected sources or infected destinations;
- whilst animals on the source premises were estimated to be in their infectious period; and

- whilst animals on the destination premises were estimated to have first been infected.

For each IP, if a single such possibly infectious trace was available then this was assumed to be the source for this premises, irrespective of genomic or other analyses. If multiple possibly infectious traces were available then the trace with maximal combine epidemiological temporal, spatial and genomic likelihood was assumed to be the source of infection on this IP. For every other IP, if the proposed most likely source based on the original method by [6] was >20 km from the destination and by definition there did not exist a possibly infectious trace linking these premises, 100% tracing sensitivity was assumed (for the purposes of this analysis) and given that 'local spread' (see definition in Supplementary Materials S1, Table A) would be considered highly unlikely to occur over such a distance, the IP with the maximal combined epidemiological temporal, spatial and genomic likelihood was assumed to be the source of infection on this IP.

Temporal likelihood was estimated as described above [6]. Spatial likelihood was estimated based on the spatial kernel described for the 2001 outbreak of foot and mouth disease (FMD) in the Netherlands [13]:

$$k_{d_{ij}} = \frac{k_0}{1 + \left(d_{ij}/d_0\right)^{\propto}}$$

where d_{ij} is the Euclidean distance between the premises, k_0 represents the probability of infection per day of a destination premises contiguous with an infected source, d_0 and α are shape and scale parameters that determine the height and decay rate of the transmission kernel. Here, we assumed the kernel height parameter k_0 = 0.0022 day^{-1}, with spatial risk decaying mostly by 3 km (d_0 = 0.9 and α = 2.3) following parameters fit to data from the 2001 outbreak of FMD in The Netherlands [13].

Genomic likelihood was estimated as the sum of the probability of the observed changes across the whole genomes sampled at the proposed source and destination, given the time difference between sampling at each (Δt), assuming the two-parameter K80 model [14], such that a nucleotide base in the viral sequence (x) mutates to a different nucleotide base (y ≠ x) within a specified interval of time with probability:

$$P_{\mu_1,\mu_2}(y|x,\Delta t)=\begin{cases} 0.25+0.25e^{-4\mu_2\Delta t} - 0.5e^{-2(\mu_1+\mu_2)\Delta t}, & \text{for a transition} \\ 0.25 - 0.25e^{-4\mu_2\Delta t} \quad , & \text{for a transversion} \end{cases}$$

where μ_1 and μ_2 are the rates of transition and transversion, here inputted as 2.52×10^{-5} and 1.80×10^{-6}, respectively, based on those reported for the 2010 outbreak of FMD in Miyazaki Prefecture of Japan [15].

Whilst, a nucleotide base in the viral sequence (*x*) does not mutate (*y* = *x*) over the specified time interval with probability:

$$P_{\mu_1,\mu_2}(y|x,\Delta t)=0.25+0.25e^{-4\mu_2\Delta t}+0.5e^{-2(\mu_1+\mu_2)\Delta t}, \qquad \text{for no mutation}$$

Sampled Ancestors [16]

Sampled Ancestors (version 1.1.7) is a Bayesian reversible-jump MCMC algorithm that extends the transmission birth-death skyline model implemented in BEAST version 2.4.7 [17] so sampled individuals can remain extant in the tree and be direct ancestors of other sampled individuals. Sampled Ancestors was originally developed for inference on a cluster of HIV-1 subtype B from 62 human patients in the United Kingdom. Two inferred parameters control the prevalence of sampled ancestors (i.e. IPs that infect other IPs, rather than dead ends in the transmission tree): the sampling proportion of such sampled ancestors (i.e. the detection rate) and the removal probability limiting how many of the sampled ancestors remain able to cause further infections after they are first detected. Using the skyline process, the sampling proportion was inferred separately in distinct periods of time before and after detection of the outbreaks on day 21. A Beta(1,4) prior was placed on the rate of recovery to represent a relatively short infectious duration at the premises level (centred on 5 days) to reflect the speed that most premises are culled following detection. The prior for the basic reproductive rate (R_0) was assumed to be Log Normal with mean of 2 and (95% probability density: 0.66, 4.7). The tree height prior was assumed to be Log Normal with mean of 130 days (95% probability density: 43, 306 days). Flat priors were placed on all other inferred parameters. MCMC chains were run for 20 million cycles (10% discarded as burn-in and thinning by 1000), taking around 2 hrs on a 2.6 GHz laptop with 32 GB of available RAM. Chains were checked with Tracer version 1.6 (http://tree.bio.ed.ac.uk/software/tracer) for required length of burn-in, no obvious trends or severe autocorrelation and that effective sample sizes (ESS) for all inferred parameters were >200. Tree annotator (version 2.4.7) was used to construct the 50% consensus maximum clade creditability tree and the accuracy of the tree and inferred parameters estimated using R (version 3.4.1).

Outbreaker [18]

The Outbreaker model (version 1.1-8) for partial Bayesian MCMC inference of the transmission tree of densely sampled outbreaks was run in R (version 3.4.1) on a parallel computing cluster to facilitated checking for convergence, with 2 chains (1 per compute node, taking 13 to 15 hours to run, dependant on the number of observed samples, on each 2.6 GHz node with 16 GB of available RAM) for 2 million MCMC iterations discarding the first 10% as burn-in as informed by the Gelman

and Rubin's shrink factor, and thinning the chains by 10,000 based on assessment of autocorrelation. This model implements a generational clock for the accumulation of genetic diversity, without attempting to incorporate within-host evolution. Given the short timescale considered between pairs of sequences, the probability of reverse mutations were considered negligible and no sites were removed for reasons of being considered under strong selection (such as immune epitopes) given the lack of evidence of positive selection occurring at an amino acid level anywhere in the genome in a previous phylogenetic analysis [19].

Based on empirical data [20], fixed model inputs included discretised truncated gamma distributions representing the generation time distribution (ω, the time interval between primary and secondary infections) as ω ~ Gamma(shape = 3.0, scale = 2.7) with mean = 8.1, SD = 4.7; and the infection to sampling interval distribution (*f*, the time difference between infection and sampling) as *f* ~ Gamma(9.13, 1.52) with mean = 13.9, SD=4.6; both truncated at 40 days. A flat Beta(1,1) prior was placed on the sampling density. The initialising tree was set with sources selected randomly from amongst those estimated to hold infectious animals at the estimated time of exposure of each farm. If there were no potential sources at the estimated time of exposure of a farm it was initialised with a value to represent seeding from a non-observed case. The following were imputed: the source of infection for each IP, the timing of infection of each IP, the number of generations between each source and infected IP, the proportion of cases observed and the rates of transitions and transversions (μ_1 and μ_2, respectively).

Lau's joint Bayesian inference [21]

Lau's joint Bayesian MCMC inference of the transmission tree with data augmentation for unobserved sequences and further parameters was compiled from C++ source code and run on a parallel computing cluster with 4 chains for 100,000 MCMC iterations discarding the first 50,000 iterations as burn-in until convergence was reached assessed with Gelman and Rubin's shrink factor, and thinning the chains by 100 based on assessment of autocorrelation. Each chain took between 3 to 9 hours to run dependant on the number of observed samples (*n* ranging from 98 to 298) on each 2.6 GHz compute node with 16 GB of available RAM). Sensitivity analyses (data not shown) demonstrated that these run dimensions gave similar results (no inferential difference or accuracy change) compared to single longer runs of up to 1,000,000 MCMC iterations with 10% burn-in and thinning by 100.

The MCMC was initialised with a transmission tree with initial sources selected randomly from amongst those estimated to hold infectious animals at the estimated time of exposure of each farm.

If there were no potential sources at the estimated time of exposure of a farm it was initialised with a value to represent seeding from a non-observed case. In this study, given all cases in the cluster were considered to have been observed (albeit in certain runs not all with available sequence data), this would represent seeding from elsewhere. The initiating single universal master sequence (G_M) was assumed to be the earliest sampled sequence. A power law kernel was used to represent the spatial relationship between premises of the form:

$$k_{d_{ij}} = \frac{1}{1 + {d_{ij}}^{\kappa}}$$

where d_{ij} is the Euclidean distance between the premises and κ is an inferred parameter.

All unobserved parameters were given uninformative flat priors and the following were imputed: timing of exposure for each premises, the sources of infection for each premises, the universal master sequence (G_M), the sequence on each premises at each transmission event (including premises for which sequence data was unavailable), the power of the spatial transmission kernel (κ), the mean and variance of the latent period, the mean period from Day of onset to Last day of culling (approximately the farm-level infectious period), primary and secondary transmission rates, and the rates of transitions and transversions (μ_1 and μ_2, respectively).

<u>BeastLier [22]</u>

Hall et al's algorithm was implemented in BEAST version 1.8.4 [23] to fully explore the space of transmission trees and nested phylogenies based on specifically designed moves devised to alter the phylogeny and node partitions in such a way that inferred transmission tree structure is maintained. The HKY substitution model [24] was used with strict molecular clock and a power law kernel to representing the spatial relationship between sampled premises. MCMC chains of 50 million cycles (discarding 20% burn-in, and thinning by 4000) were initiated with a starting transmission tree randomly specified as for the Lau model (detailed above), with a random starting phylogeny nested within the transmission tree. A Gamma(7, 1) prior was assumed for the infectious duration (median = 6.7 days, 95% probability density: 2.8, 13.0 days) and a Gamma(5, 1) was assumed for the latent period (median = 4.6 days, 95% probability density: 1.6, 10.2 days) [25,26]. Non-informative priors were placed on the date of the index infection and all other priors. Each chain took approximately 3 hours to run.

Structured COalescent Transmission Tree Inference, SCOTTI [27]

The SCOTTI approximate structured coalescent framework (version 1.1.0) was used to infer a tree of transmission events between populations of pathogens on each infected premises. In the coalescent process, transmission events were represented as migration rates between distinct pathogen populations [27]. SCOTTI was implemented in BEAST version 2.4.7 [17] with the HKY substitution model [24] with 2 independent chains of 2 million MCMC iterations each (10% discarded as burn-in, and thinned by 1000), taking between 16 to 29 hours for each chain to run dependant on the number of observed samples. Data were collated pre- and post-model runs with Python scripts distributed with the open source SCOTTI package, model outputs were then manipulated and analysed in R version 3.4.1 [8]. Convergence was confirmed using Tracer v1.6 (http://tree.bio.ed.ac.uk/software/tracer/, last accessed 31 Oct 2017). Chains for inferred parameters were thinned by 200 and posterior transmission tree chains were thinned by 400. The maximum number of hosts was set to 10 times the number of sequences available to allow for unobserved IPs, observed IPs for which genomic data was missing and seeding from external clusters. SCOTTI does not allow explicit representation of observed hosts missing genomic data, so when undertaking accuracy assessment, it was not possible to identify those infected from unobserved IPs, observed IPs for which genomic data was missing and seeding from external clusters, as all of these occurrences are represented in model outputs as infection from non-sampled hosts. Given typically very low and severely positively skewed model support, the likelihoods were non-parametrically rescaled between 0 and 1 for interpretation. All unobserved parameters were given uninformative flat priors and the following were imputed: the sources of infection for each premises, the mutation rate, ratio of transitions to transversions, the HKY frequency parameters (π_A, π_C, π_G, π_T), the total outbreak size (as the migratory model number of populations) in terms of number of IPs including unobserved cases (allowing estimation of the proportion of undetected cases), the within-host effective viral population size (migratory population size) and the rate of transmission events (as the migration rate).

Phybreak [28]

For each simulation, the Phybreak partial Bayesian MCMC inference incorporating within-host evolution, which follows earlier work by [29], was run in R (version 3.4.1) for 2 independent chains each consisting of 10,000 burn-in and a further 50,000 MCMC iterations for the sample, thinned by 100. The generation time distribution (ω) was assumed to follow a Gamma distribution with fixed shape parameter of 3 and a mean that is estimated, here initialised at 8.1 days with SD of ω of 4.7 days as informed by empirical data [20], considering that it would be unlikely to have excellent prior

information on this distribution in an outbreak in a previously free country. The sampling time interval (τ) period between infection and sampling was assumed to be Gamma distributed with fixed shape = 9.13 and a mean that is estimated, here initialised at 13.9 days with SD of τ of 4.6 days, again informed by empirical data [20], considering that this distribution could be estimated with relatively high accuracy soon after commencement of an outbreak based on data on timing of sampling and Day of onset of clinical signs. The within-host coalescent process was assumed to occur after transmission (i.e. in the infected host) with a linearly increasing slope of the effective pathogen population size with time after infection. The mutation rate (μ) was based on a site-homogeneous Jukes-Cantor model. Uninformative priors, as detailed in [30], were placed on all unknown parameters: the structure of the transmission tree, timing of infection at each node in the tree, the mean of the generation time and sampling time distributions, the slope of the within-host coalescent process and the mutation rate. Each chain took between 8 to 32 hours to run dependant on the number of observed samples.

TransPhylo [31]

The phylogenetic tree used to initiate the TransPhylo analysis was inferred in BEAST version 2.4.7 [17] with the HKY substitution model [24] and a strict molecular clock, run for 10 million MCMC cycles (20% discarded as burn-in) and Tree annotator (version 2.4.7) was used to construct the 50% consensus maximum clade creditability tree. TransPhylo's Bayesian reversible-jump MCMC algorithm for partially sampled and ongoing outbreaks was run in R (version 3.4.1) for 1 million cycles (10% discarded as burn-in, thinned by 1000), to infer the transmission tree based on the given phylogeny. As described above, based on empirical data [20], the generation time distribution was assumed to follow a Gamma(3.1, 2.61) and the delay in sampling was assumed to have follow a Gamma(2.70, 2.90). The starting value of the inferred within-host coalescent parameter (N_e*g) was set to 100 to represent a within-host population size of N_e = 100 and a generation time (g) of 1 day. The sampling proportion was fixed at 99%. Each chain took approximately 9 hours to run.

Outbreaker2 [32]

The Outbreaker2 model (version 1.0.1) was run in R (version 3.4.1). The parameterisation was exactly as described above for the Outbreaker model [18], except that only a single mutation rate (μ) is inferred (as that is the only option available) and known contact-tracing data was incorporated into the analysis and the contact reporting coverage inferred utilising a flat Beta(1,1) prior distribution.

Table S2.1: Comparison of the data used for each outbreak transmission network inference, by model.

Method	Genomic sequence data (fasta)	Date of sampling	Estimated date of onset of clinical signs	Estimated sampling delay	Date of culling / removal / Estimated last day of infection	Estimated generation time distribution	Estimated sampling proportion	Spatial coordinates of farms	Contact-tracing data
Cottam et al., 2008	+	+	+	±	+	-	-	-	-
Cottam et al. (modified)	+	+	+	±	+	-	-	+	+
Gavryushkina et al., 2014 (SA)	+	+	-	-	-	-	+	-	-
Jombart et al., 2014 (Outbreaker)	+	+	-	+	-	+	+	-	-
Lau et al., 2015	+	+	+	±	+	-	-	+	-
Hall et al., 2015 (BeastLier)	+	+	-	-	+	-	-	+	-
De Maio et al., 2016 (SCOTTI)	+	+	+	±	+	-	-	-	-
Klinkenberg et al., 2017 (Phybreak)	+	+	-	+	-	+	-	-	-
Didelot et al., 2017 (TransPhylo)	+	+	-	+	-	+	+	-	-
Campbell et al., 2018 (Outbreaker2)	+	+	-	+	-	+	+	-	+

± = indirectly required (i.e., sampling delay is estimated from estimated date of onset and date of sampling). SA = Sampled Ancestors.

S3: Detailed results

Table S3.1 Comparison of the accuracy of inferences of transmission network models over six simulated outbreaks of foot-and-mouth disease (FMD) in Australia, detailed by run.

A) Genomic data available for all infected premises.

**Model**	**Run**	**Accuracy**[a] Overall (%)	>50% support (%)	>80% support (%)
Cottam et al., 2008	1	20/41[b] (49)	13/19 (68)	7/9 (78)
	2	28/69[b] (41)	14/33 (42)	7/15 (47)
	3	56/97[b] (58)	35/49 (71)	18/20 (90)
	4	60/99[b] (61)	34/50 (68)	17/20 (85)
	5	65/155[b] (42)	37/76 (49)	12/32 (38)
	6	144/297[b] (48)	99/143 (69)	54/62 (87)
Cottam et al. (modified)	1	30/41[b] (73)	15/19 (79)	8/9 (89)
	2	45/69[b] (65)	24/33 (73)	8/15 (53)
	3	74/97[b] (76)	41/49 (84)	19/20 (95)
	4	67/99[b] (68)	40/50 (80)	17/20 (85)
	5	97/155[b] (63)	52/76 (68)	19/32 (59)
	6	195/297[b] (66)	116/142 (82)	55/62 (89)
Gavryushkina et al., 2014 (Sampled Ancestors)	1	7/15[c] (47)	3/5 (60)	2/3 (67)
	2	4/38[c] (11)	2/21 (10)	1/8 (12)
	3	26/76[c] (34)	19/39 (49)	10/16 (62)
	4	12/44[c] (27)	7/24 (29)	7/8 (88)
	5	33/110[c] (30)	19/56 (34)	11/23 (48)
	6	41/228[c] (18)	22/105 (21)	16/43 (36)
Jombart et al., 2014 (Outbreaker)	1	16/42 (38)	16/40 (40)	15/34 (44)
	2	23/70 (33)	22/60 (37)	16/38 (42)
	3	36/98 (37)	36/89 (40)	35/78 (45)
	4	38/100 (38)	32/70 (46)	25/50 (50)
	5	61/156 (39)	60/153 (39)	51/125 (41)
	6	128/298 (43)	127/271 (47)	110/185 (59)
Lau et al., 2015	1	27/42 (64)	22/26 (85)	17/18 (94)
	2	47/70 (67)	46/61 (75)	36/46 (78)
	3	77/98 (79)	70/82 (85)	60/64 (94)
	4	76/100 (76)	75/93 (81)	60/69 (87)
	5	115/156 (74)	103/124 (83)	80/84 (95)
	6	217/298 (73)	203/250 (81)	153/164 (93)
Hall et al., 2015 (BeastLier)	1	13/42 (31)	9/21 (43)	4/9 (44)
	2	17/70 (24)	11/35 (31)	5/14 (36)
	3	13/98 (13)	8/49 (16)	3/20 (15)
	4	21/100 (21)	15/50 (30)	7/21 (33)
	5	26/156 (17)	13/78 (17)	7/32 (22)
	6	28/298 (9)	10/149 (7)	5/60 (8)

De Maio et al., 2016 (ScoTTI)	1	17/42 (40)	12/21 (57)	7/9 (78)
	2	37/70 (53)	25/35 (71)	13/14 (93)
	3	39/98 (40)	35/49 (71)	16/20 (80)
	4	52/100 (52)	37/50 (74)	20/20 (100)
	5	89/156 (57)	61/78 (78)	31/32 (97)
	6	149/298 (50)	112/149 (75)	57/60 (95)
Klinkenberg et al., 2017 (Phybreak)	1	13/42 (31)	7/12 (58)	2/3 (67)
	2	23/70 (33)	12/23 (52)	4/6 (67)
	3	45/98 (46)	24/36 (67)	16/18 (89)
	4	58/100 (58)	34/41 (83)	13/13 (100)
	5	64/156 (41)	35/47 (74)	20/24 (83)
	6	160/298 (54)	110/131 (84)	57/59 (97)
Didelot et al., 2017 (TransPhylo)	1	1/42 (2)	1/21 (5)	1/9 (11)
	2	6/70 (9)	4/35 (11)	1/14 (7)
	3	4/98 (4)	3/49 (6)	3/20 (15)
	4	9/100 (9)	5/50 (10)	2/20 (10)
	5	5/156 (3)	3/78 (4)	0/32 (0)
	6	4/298 (1)	2/149 (1)	2/60 (3)
Campbell et al., 2018 (Outbreaker2)	1	12/42 (29)	9/26 (35)	8/20 (40)
	2	24/70 (34)	19/43 (44)	18/29 (62)
	3	41/98 (42)	39/77 (51)	32/57 (56)
	4	36/100 (36)	31/64 (48)	23/48 (48)
	5	48/156 (31)	31/121 (32)	24/79 (33)
	6	103/298 (35)	101/250 (40)	85/141 (60)

IP = infected premises. [a] Accuracy was defined as the proportion of IPs for which the model-predicted most likely source (highest likelihood or most posterior support) was the true source. The denominator for accuracy at >50% and >80% support includes only those IPs for which the model-predicted most likely source attained that level of likelihood or posterior support. 0/0 represents a model run where none of the IPs had this level of support. [b] Root fixed based on best guess, so denominator is reduced by 1. [c] Not all IPs detected as having sampled ancestors.

B) Genomic data missing for 50% of infected premises.

Model	Run	Accuracy[a] Overall (%)	>50% support (%)	>80% support (%)
Jombart et al., 2014 (Outbreaker)	1	8/42 (19)	5/23 (22)	5/15 (33)
	2	9/70 (13)	9/39 (23)	8/27 (30)
	3	17/98 (17)	15/52 (29)	10/36 (28)
	4	13/100 (13)	9/43 (21)	7/24 (29)
	5	2/156 (1)	2/45 (4)	1/10 (10)
	6	6/298 (2)	1/12 (8)	1/7 (14)
Lau et al., 2015	1	16/42 (38)	8/16 (50)	7/12 (58)
	2	21/70 (30)	15/25 (60)	8/9 (89)
	3	57/98 (58)	27/34 (79)	10/12 (83)
	4	55/100 (55)	42/62 (68)	19/22 (86)
	5	45/156 (29)	30/74 (41)	19/39 (49)
	6	125/298 (42)	90/145 (62)	49/60 (82)
De Maio et al., 2016 (ScoTTI)	1	8/21[b] (38)	5/11 (45)	2/5 (40)
	2	12/35[b] (34)	6/18 (33)	6/7 (86)
	3	11/49[b] (22)	10/25 (40)	6/10 (60)
	4	14/50[b] (28)	12/25 (48)	7/10 (70)
	5	14/78[b] (18)	13/39 (33)	7/16 (44)
	6	34/149[b] (23)	27/75 (36)	9/30 (30)
Klinkenberg et al., 2017 (Phybreak)	1	5/42 (12)	2/2 (100)	0/0
	2	11/70 (16)	1/2 (50)	0/0
	3	14/98 (14)	3/3 (100)	0/0
	4	17/100 (17)	6/9 (67)	0/0
	5	19/156 (12)	5/10 (50)	1/3 (33)
	6	67/298 (22)	19/24 (79)	4/4 (100)
Campbell et al., 2018 (Outbreaker2)	1	3/42 (7)	0/0	0/0
	2	1/70 (1)	0/0	0/0
	3	5/98 (5)	4/49 (8)	0/20 (0)
	4	2/100 (2)	0/0	0/0
	5	3/156 (2)	0/0	0/0
	6	7/298 (2)	0/1 (0)	0/0

IP = infected premises. [a] Accuracy was defined as the proportion of IPs for which the model-predicted most likely source (highest likelihood or most posterior support) was the true source. The denominator for accuracy at >50% and >80% support includes only those IPs for which the model-predicted most likely source attained that level of likelihood or posterior support. 0/0 represents a model run where none of the IPs had this level of support. [b] SCOTTI only outputs proposed ancestors for those IPs with genomic data available.

Table S3.2 Comparison of the accuracy of inferred times of exposure for infected premises in six simulated outbreaks of foot-and-mouth disease in Australia, by transmission network model.

Model	Run	Coverage[a]	Posterior median bias in inferred time of first exposure, in days (95% HPD)
Jombart et al., 2014 (Outbreaker)	1	3/42 (0.07)	11.0 (1.1, 30.0)
	2	6/70 (0.09)	11.0 (-6.3, 27.3)
	3	3/98 (0.03)	12.0 (-2.0, 32.3)
	4	5/100 (0.05)	10.0 (-0.1, 28.6)
	5	4/156 (0.03)	12.0 (-3.1, 32.0)
	6	4/298 (0.01)	11.0 (1.0, 22.6)
Lau et al., 2015	1	35/42 (0.83)	0.1 (-7.5, 0.9)
	2	52/70 (0.74)	-0.9 (-8.5, 0.7)
	3	50/98 (0.51)	3.1 (-9.0, 4.7)
	4	77/100 (0.77)	1.4 (-7.4, 4.3)
	5	101/156 (0.65)	3.2 (-5.7, 4.6)
	6	219/298 (0.73)	1.4 (-8.9, 3.8)
Hall et al., 2015 (BeastLier)	1	6/42 (0.14)	9.7 (-31.2, 21.0)
	2	30/70 (0.43)	7.1 (-11.0, 18.3)
	3	21/98 (0.21)	8.2 (-9.8, 21.1)
	4	39/100 (0.39)	6.5 (-6.2, 18.8)
	5	18/156 (0.12)	9.5 (-14.0, 26.4)
	6	69/298 (0.23)	6.8 (-12.7, 19.2)
Klinkenberg et al., 2017 (Phybreak)	1	32/42 (0.76)	0.21 (-12.1, 18.2)
	2	50/70 (0.71)	1.85 (-10.0, 13.2)
	3	70/98 (0.71)	2.58 (-10.4, 19.2)
	4	79/100 (0.79)	1.05 (-8.0, 15.2)
	5	126/156 (0.81)	1.38 (-10.7, 16.5)
	6	257/298 (0.86)	0.76 (-8.8, 11.0)
Didelot et al., 2017 (TransPhylo)	1	15/42 (0.36)	-1.92 (-26.7, 43.7)
	2	20/70 (0.29)	-2.29 (-17.3, 62.5)
	3	44/98 (0.45)	4.56 (-18.7, 23.7)
	4	42/100 (0.42)	3.12 (-13.6, 37.9)
	5	5/156 (0.03)	10.01 (-52.6, 43.2)
	6	1/298 (0.00)	-18.08 (-54.2, 103.8)
Campbell et al., 2018 (Outbreaker2)	1	38/42 (0.90)	-2.0 (-19.9, 2.0)
	2	58/70 (0.83)	-2.0 (-25.0, 0.0)
	3	86/98 (0.88)	-2.0 (-21.6, 1.0)
	4	85/100 (0.85)	-2.0 (-20.5, 1.0)
	5	137/156 (0.88)	-2.0 (-20.2, 1.0)
	6	260/298 (0.87)	-2.0 (-20.6, 1.0)

[a] Coverage is the proportion of infected premises whose 95% Highest Posterior Density (HPD) region for the inferred infection time encompassed the true (simulated) value.

Table S3.3 Sensitivity Analysis: Comparison of the accuracy of inferences of transmission network models for modified parameterisations of simulated outbreaks of foot-and-mouth disease (FMD) in Australia.

**Model**	**Run**	**Accuracy**[a] Overall (%)	>50% support (%)	>80% support (%)
Cottam et al., 2008	baseline	56/97[b] (58)	35/49 (71)	18/20 (90)
	non-spatial	56/97[b] (58[c])	35/49 (71[c])	18/20 (90[c])
	fast clock	60/97[b] (62)	33/50 (66)	11/19 (58)
Cottam et al. (modified)	baseline	66/97[b] (68)	36/49 (73)	18/20 (90)
	non-spatial	48/97[b] (49)	24/49 (49)	15/20 (75)
	fast clock	77/97[b] (79)	43/50 (86)	14/19 (74)
Gavryushkina et al., 2014 (Sampled Ancestors)	baseline	26/76[d] (34)	19/39 (49)	10/16 (62)
	non-spatial	27/77[d] (35)	19/38 (50)	10/16 (62)
	fast clock	2/4[d] (50)	1/1 (100)	1/1 (100)
Jombart et al., 2014 (Outbreaker)	baseline	36/98 (37)	36/89 (40)	35/78 (45)
	non-spatial	39/98 (40)	39/97 (40)	37/81 (46)
	fast clock	40/98 (41)	40/98 (41)	37/91 (41)
Lau et al., 2015	baseline	77/98 (79)	70/82 (85)	60/64 (94)
	non-spatial	69/98 (70)	56/62 (90)	47/50 (94)
	fast clock	77/98 (79)	71/82 (87)	58/64 (91)
Hall et al., 2015 (BeastLier)	baseline	13/98 (13)	8/49 (16)	3/20 (15)
	non-spatial	8/98 (8)	7/49 (14)	1/21 (5)
	fast clock	7/98 (7)	7/49 (14)	2/20 (10)
De Maio et al., 2016 (ScoTTI)	baseline	39/98 (40)	35/49 (71)	16/20 (80)
	non-spatial	41/98 (42)	33/49 (67)	17/20 (85)
	fast clock	4/98 (4)	3/49 (6)	3/20 (15)
Klinkenberg et al., 2017 (Phybreak)	baseline	45/98 (46)	24/36 (67)	16/18 (89)
	non-spatial	44/98 (45)	24/36 (67)	16/18 (89)
	fast clock	47/98 (48)	33/52 (63)	12/14 (86)
Didelot et al., 2017 (TransPhylo)	baseline	4/98 (4)	3/49 (6)	3/20 (15)
	non-spatial	6/98 (6)	1/50 (2)	1/20 (5)
	fast clock	6/98 (6)	6/49 (12)	2/20 (10)
Campbell et al., 2018 (Outbreaker2)	baseline	41/98 (42)	39/77 (51)	32/57 (56)
	non-spatial	41/98 (42)	40/77 (52)	33/57 (58)
	fast clock	35/98 (36)	34/93 (37)	34/93 (37)

IP = infected premises; 'non-spatial' = randomised spatial coordinates of each infected premises; 'fast clock' = rates of transitions and transversions increased by a factor of 10 compared to baseline. [a] Accuracy was defined as the proportion of IPs for which the model-predicted most likely source (highest likelihood or most posterior support) was the true source. The denominator for accuracy at >50% (i.e., consensus) and >80% support

includes only those IPs for which the model-predicted most likely source attained that level of likelihood or posterior support. 0/0 represents a model run where none of the IPs had this level of support. [b] Root fixed based on best guess, so denominator is reduced by 1. [c] Cottam et al.'s original method did not account for spatial relationships between points, so this is the same as for the baseline parameterisation. [d] Not all IPs detected as sampled ancestors.

References

1 Bradhurst, R. A., Roche, S. E., East, I. J., Kwan, P. & Garner, M. G. A hybrid modeling approach to simulating foot-and-mouth disease outbreaks in Australian livestock. *Frontiers in Environmental Science* **3**, 17 (2015).

2 Ratmann, O. *et al.* Phylogenetic tools for generalized HIV-1 epidemics: findings from the PANGEA-HIV methods comparison. *Mol. Biol. Evol.* **34**, 185-203 (2016).

3 Rambaut, A. & Grass, N. C. Seq-Gen: an application for the Monte Carlo simulation of DNA sequence evolution along phylogenetic trees. *Bioinformatics* **13**, 235-238 (1997).

4 Animal Health Australia. *Disease strategy: Foot-and-mouth disease (Version 3.3). Australian Veterinary Emergency Plan (AUSVETPLAN), Edition 3, Standing Council on Primary Industries, Canberra, ACT.*, (2012).

5 Gibbens, J. C. *et al.* Descriptive epidemiology of the 2001 foot-and-mouth disease epidemic in Great Britain: the first five months. *Vet. Rec.* **149**, 729-743 (2001).

6 Cottam, E. M. *et al.* Integrating genetic and epidemiological data to determine transmission pathways of foot-and-mouth disease virus. *Proc. R. Soc. Lond. B Biol. Sci.* **275**, 887-895 (2008).

7 Clement, M., Posada, D. & Crandall, K. A. TCS: a computer program to estimate gene genealogies. *Mol Ecol* **9**, 1657-1659 (2000).

8 R Core Team. (2018).

9 Csardi, G. & Nepusz, T. The igraph software package for complex network research. *InterJournal* **Complex Systems**, 1695 (2006).

10 Juleff, N. *et al.* Accumulation of nucleotide substitutions occurring during experimental transmission of foot-and-mouth disease virus. *J. Gen. Virol.* **94**, 108-119 (2013).

11 Firestone, S. M., Christley, R. M., Ward, M. P. & Dhand, N. K. Adding the spatial dimension to the social network analysis of an epidemic: Investigation of the 2007 outbreak of equine influenza in Australia. *Prev. Vet. Med.* **106**, 123-135, doi:10.1016/j.prevetmed.2012.01.020 (2012).

12 Shirley, M. D. F. & Rushton, S. P. Where diseases and networks collide: lessons to be learnt from a study of the 2001 foot-and-mouth disease epidemic. *Epidemiol Infect* **133**, 1023-1032 (2005).

13 Backer, J., Hagenaars, T., Nodelijk, G. & Van Roermund, H. Vaccination against foot-and-mouth disease I: Epidemiological consequences. *Prev. Vet. Med.* **107**, 27-40 (2012).

14 Kimura, M. Evolutionary rate at the molecular level. *Natur* **217**, 624-626 (1968).

15 Nishi, T. *et al.* Genome variability of foot-and-mouth disease virus during the short period of the 2010 epidemic in Japan. *Vet Microbiol* **199**, 62-67 (2017).

16 Gavryushkina, A., Welch, D., Stadler, T. & Drummond, A. J. Bayesian inference of sampled ancestor trees for epidemiology and fossil calibration. *PLoS Comput. Biol.* **10**, e1003919 (2014).

17 Bouckaert, R. *et al.* BEAST 2: a software platform for Bayesian evolutionary analysis. *PLoS Comput. Biol.* **10**, e1003537 (2014).

18 Jombart, T. *et al.* Bayesian reconstruction of disease outbreaks by combining epidemiologic and genomic data. *PLoS Comput. Biol.* **10**, e1003457 (2014).

19 Cottam, E. M. *et al.* Molecular epidemiology of the foot-and-mouth disease virus outbreak in the United Kingdom in 2001. *J. Virol.* **80**, 11274-11282 (2006).
20 Haydon, D. T. *et al.* The construction and analysis of epidemic trees with reference to the 2001 UK foot-and-mouth outbreak. *Proc. R. Soc. Lond. B Biol. Sci.* **270**, 121-127, doi:10.1098/rspb.2002.2191 (2003).
21 Lau, M. S., Marion, G., Streftaris, G. & Gibson, G. A systematic Bayesian integration of epidemiological and genetic data. *PLoS Comput. Biol.* **11**, e1004633 (2015).
22 Hall, M., Woolhouse, M. & Rambaut, A. Epidemic reconstruction in a phylogenetics framework: transmission trees as partitions of the node set. *PLoS Comput. Biol.* **11**, e1004613 (2015).
23 Drummond, A. J., Suchard, M. A., Xie, D. & Rambaut, A. Bayesian phylogenetics with BEAUti and the BEAST 1.7. *Mol. Biol. Evol.* **29**, 1969-1973 (2012).
24 Hasegawa, M., Kishino, H. & Yano, T.-a. Dating of the human-ape splitting by a molecular clock of mitochondrial DNA. *J Mol Evol* **22**, 160-174 (1985).
25 Mardones, F., Perez, A., Sanchez, J., Alkhamis, M. & Carpenter, T. Parameterization of the duration of infection stages of serotype O foot-and-mouth disease virus: an analytical review and meta-analysis with application to simulation models. *Vet. Res.* **41**, 45 (2010).
26 Keeling, M. J. *et al.* Dynamics of the 2001 UK foot and mouth epidemic: stochastic dispersal in a heterogeneous landscape. *Sci* **294**, 813-817 (2001).
27 De Maio, N., Wu, C.-H. & Wilson, D. J. SCOTTI: Efficient Reconstruction of Transmission within Outbreaks with the Structured Coalescent. *PLoS Comput. Biol.* **12**, e1005130 (2016).
28 Klinkenberg, D., Backer, J. A., Didelot, X., Colijn, C. & Wallinga, J. Simultaneous inference of phylogenetic and transmission trees in infectious disease outbreaks. *PLoS Comput. Biol.* **13**, e1005495 (2017).
29 Ypma, R. *et al.* Unravelling transmission trees of infectious diseases by combining genetic and epidemiological data. *Proc. R. Soc. Lond. B Biol. Sci.*, rspb20110913 (2011).
30 Klinkenberg, D., Backer, J., Didelot, X., Colijn, C. & Wallinga, J. New method to reconstruct phylogenetic and transmission trees with sequence data from infectious disease outbreaks. *bioRxiv*, 069195 (2016).
31 Didelot, X., Fraser, C., Gardy, J. & Colijn, C. Genomic infectious disease epidemiology in partially sampled and ongoing outbreaks. *Mol. Biol. Evol.* **34**, 997-1007 (2017).
32 Campbell, F. *et al.* outbreaker2: a modular platform for outbreak reconstruction. *BMC Bioinformatics* **19**, 363 (2018).
